# Anterograde irrigation - assisted ureteroscopic lithotripsy in patients with percutaneous nephrostomy

**DOI:** 10.1590/S1677-5538.IBJU.2018.0238

**Published:** 2019-04-01

**Authors:** Jemo Yoo, Seung-Ju Lee, Hyun-Sop Choe, Hee Youn Kim, Joon Ho Lee, Dong Sup Lee

**Affiliations:** 1 St. Vincent's Hospital, College of Medicine, The Catholic University of Korea

## Abstract

In complicated urinary tract infection with ureteral calculi, urinary diversion is inevitable. So, stenting or percutaneous drainage can be an option. In hemodynamically unstable patients, percutaneous drainage is superior to ureteral stenting (1). Once acute infection is controlled, definite treatment of the stone is necessary. According to a guideline, semirigid ureteroscopy is recommended for lower and mid - ureter stone and flexible ureteroscopy for upper ureter stone (2). Semi - rigid ureteroscopy can migrate stone to kidney, especially in upper ureter stone, lowering stone free rate (3). Not only flexible ureteroscopy creates additional costs but also is barely available in developing countries (4, 5). So, the authors would like to introduce anterograde irrigation - assisted ureteroscopic lithotripsy in patients with percutaneous nephrostomy.

Retrograde irrigation was connected and flowed minimally enough to secure visual field. Once stone is noted, another saline irrigation, which is placed above 40 cm over the patient is connected to nephrostomy. Retrograde irrigation is disconnected from ureteroscope and the previous connected channel on ureteroscope is opened. Actual pressure detected by barometer from the opened channel of ureteroscope is usually about 30 cmH_2_ O while anterograde irrigation is administered in maximal flow, which means fully opened anterograde irrigation is not hazardous to kidney. There was no complication in 17 patients submitted to this method.

Video shows advantages of our practice: clear visual field; reduced risk of stone migration into kidney; induced spontaneous passage of fragments without using instrumentation; and decreased operation time. In short, most of surgeons, even unexperienced, can perform an excellent procedure with less time consuming using our method.

## ARTICLE INFO

**Available at: http://www.intbrazjurol.com.br/video-section/20180238_Yoo_et_al**

**Int Braz J Urol. 2019; 45 (video #5): 406-7**
